# Supplementary material for: Phase variable colony morphotypes of Clostridioides difficile elicit distinct host responses during acute infection
Source: Infect Immun. 2026 Jun 9;94(7):e00022-26. doi: 10.1128/iai.00022-26 (PMC13367059; doi:10.1128/iai.00022-26)
Supplement: Supplemental material — Fig. S1 to S7; Tables S1 and S2. [file iai.00022-26-s0001.pdf]

SUPPLEMENTAL MATERIAL

Phase variable colony morphotypes of *Clostridioides difficile* elicit distinct host responses during acute infection

Jilarie A. Santos-Santiago [a], Nicole C. Gadda [a], Rani Sellers [b], and Rita Tamayo [a,*]

[a] Department of Microbiology and Immunology, University of North Carolina Chapel Hill School of Medicine, Chapel Hill, North Carolina, USA

[b] Department of Pathology and Laboratory Medicine, University of North Carolina at Chapel Hill School of Medicine, Chapel Hill, North Carolina, USA

* Corresponding author
E-mail: rita_tamayo@med.unc.edu (RT)

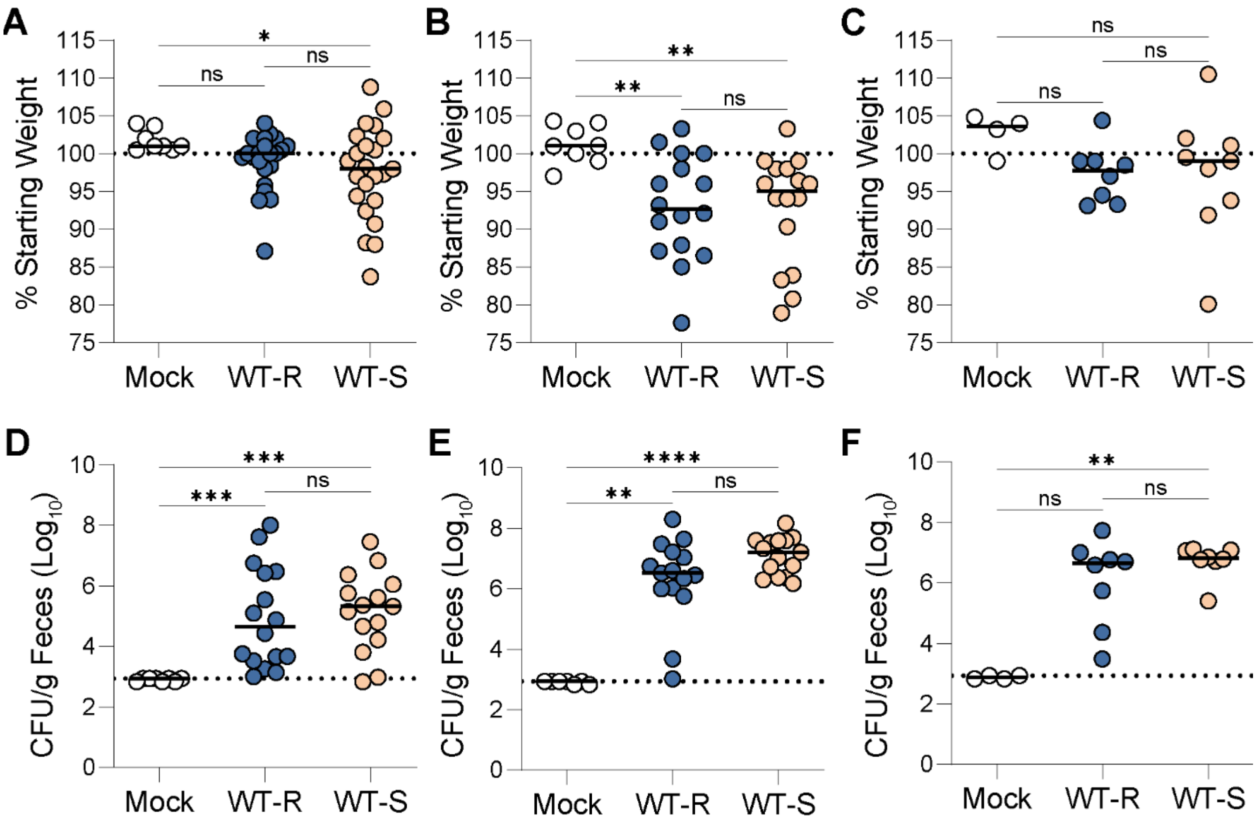

Figure S1. Weight loss and fecal burden in mice infected with rough and smooth colony isolates of wild-type *C. difficile*. Male and female C57BL/6 mice were inoculated with spores of wild-type rough (WT-R) or smooth (WT-S) colony isolates (Garrett, 2019). Mock-inoculated animals were included as controls. **(A-C)** *C. difficile*-induced weight loss on **(A)** day 1, **(B)** day 2, and **(C)** day 3 post-inoculation (p.i.). Weights are expressed as a percentage of each mouse's starting weight at the time of inoculation. **(D-F)** *C. difficile* burden in feces. Colony-forming units (CFU) in fecal samples were enumerated on **(D)** day 1, **(E)** day 2, and **(F)** day 3 p.i. The dotted line represents the limit of detection. No *C. difficile* was detected in the mock-inoculated mice. Symbols represent values from individual animals, and lines denote medians. **** p ≤ 0.0001, *** p ≤ 0.001, ** p ≤ 0.01, * p ≤ 0.05, and ns = not significant; Kruskal-Wallis test and Dunn's post-test.

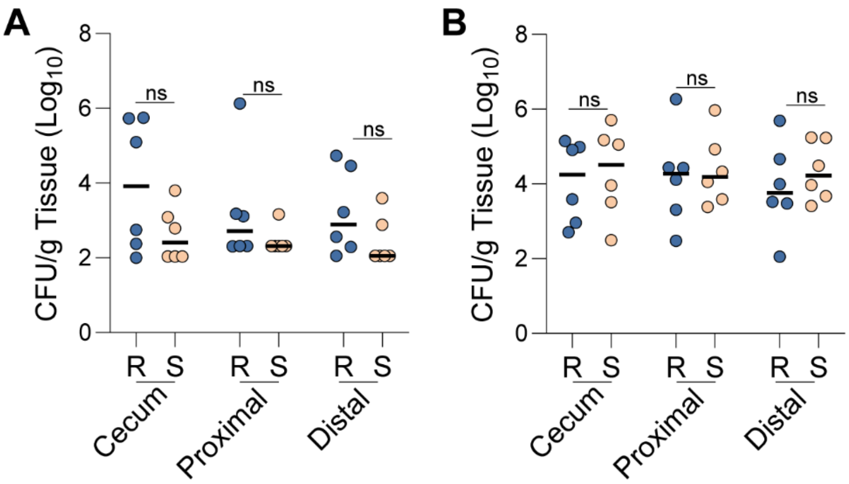

Figure S2. *C. difficile* burden in tissue. C57BL/6 mice were inoculated with spores of wild-type rough (R) or smooth (S) colony isolates. The cecum, proximal colon, and distal colon were collected on (**A**) day 1 and (**B**) day 2 p.i., and CFU were enumerated. Circles indicate values from individual mice, and lines denote medians. ns = not significant; Mann-Whitney test per tissue.

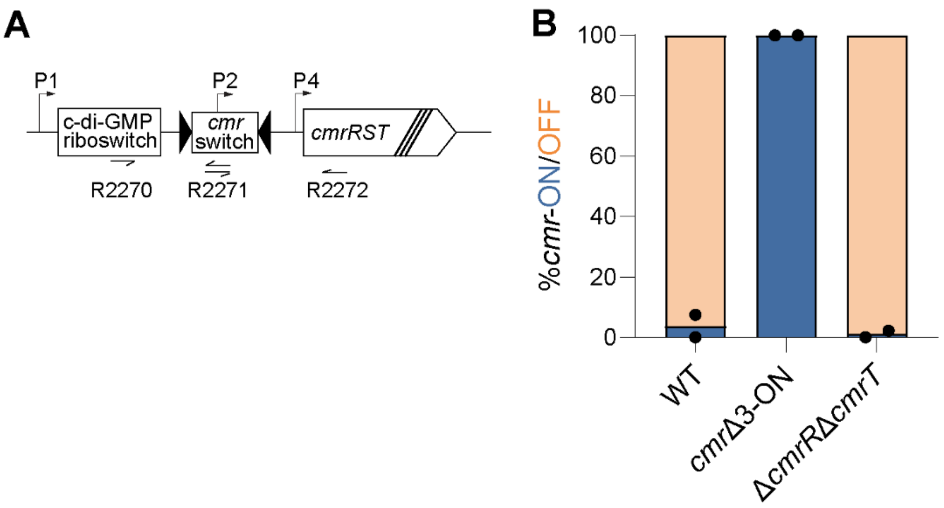

Figure S3. Quantification of the *cmr* switch orientation in the inoculums for Figure 3. (A) Diagram of the *cmrRST* locus (not to scale). The c-di-GMP riboswitch and associated promoter (P1) are indicated (Sudarsan, 2008; McKee, 2018; Garrett, 2021). Black triangles represent the inverted repeats (28 bp each) flanking the *cmr* switch (272 bp), which contains another promoter (P2) (Sekulovic, 2018; Garrett, 2021). An additional promoter (P4) associated with autoregulation by CmrR is depicted (Garrett, 2021). Three primers were used to determine the orientation of the *cmr* switch. Primers R2270 and R2271 produce an amplicon from DNA containing the *cmr* switch in the “ON” orientation; primers R2271 and R2272 yield an amplicon from DNA with the inverted *cmr* “OFF” orientation (Garrett, 2021). **(B)** Quantitative PCR of the *cmr* switch orientation in spore inoculums expressed as the percentage of the population with the *cmr*-ON (blue) or *cmr*-OFF (orange) switch orientation. Circles indicate values from independent experiments.

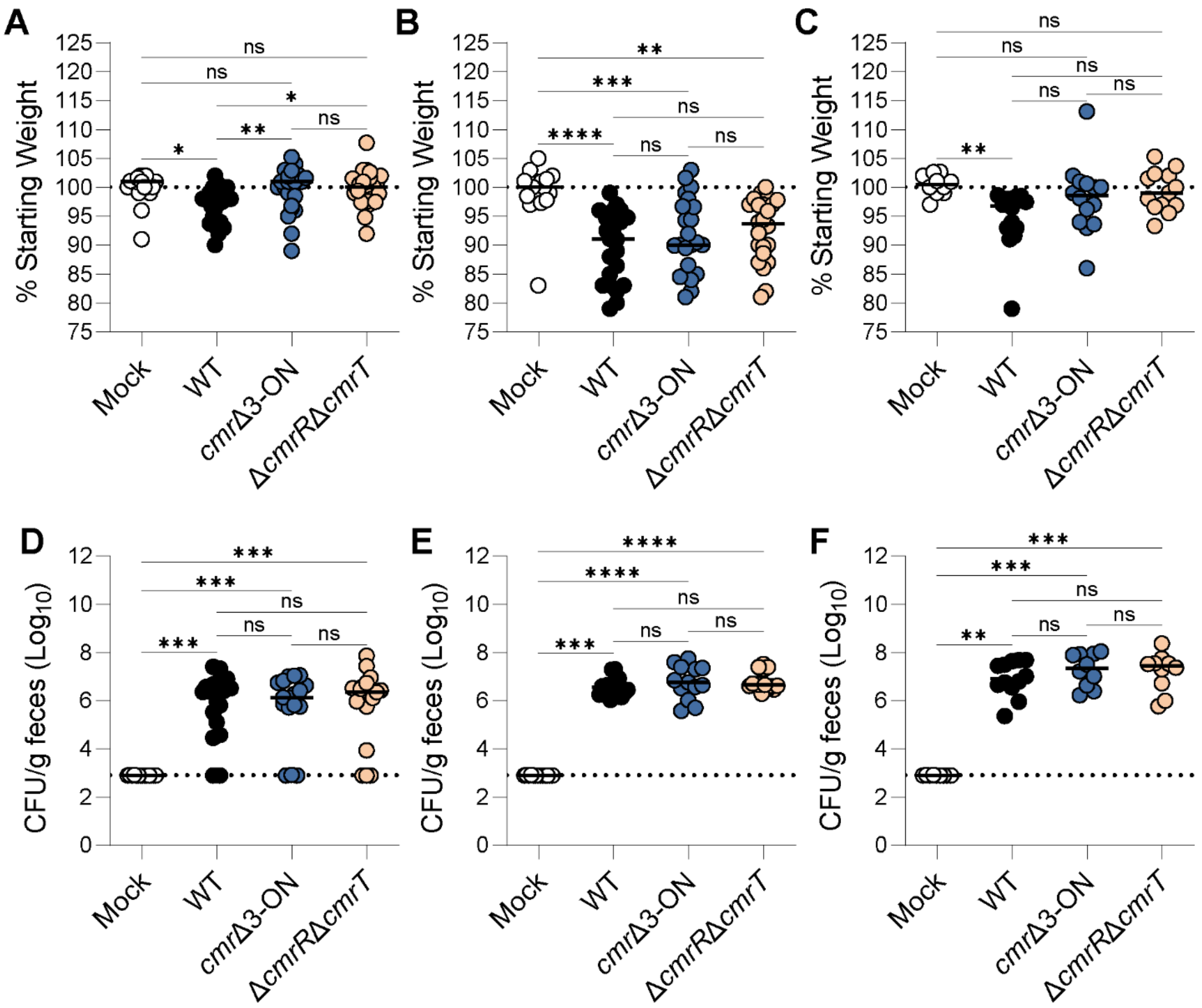

Figure S4. Weight loss and fecal burden in mice infected with phenotypically locked *C. difficile*. Male and female C57BL/6 mice were inoculated with spores of the phenotypically locked *cmr*Δ3-ON and Δ*cmrR*Δ*cmrT* mutants. Mock-inoculated animals were included as controls. **(A-C)** *C. difficile*-induced weight loss on **(A)** day 1, **(B)** day 2, and **(C)** day 3 p.i. Weights are expressed as a percentage of each mouse's starting weight at the time of inoculation. **(D-F)** *C. difficile* burden in feces. CFU in fecal samples were enumerated on **(D)** day 1, **(E)** day 2, and **(F)** day 3 p.i. The dotted lines represent the limit of detection. No *C. difficile* was detected in the mock-inoculated mice. Circles represent values from individual animals, and lines indicate medians. **** $p \leq 0.0001$, *** $p \leq 0.001$, ** $p \leq 0.01$, * $p \leq 0.05$, and ns = not significant; Kruskal-Wallis test and Dunn's post-test.

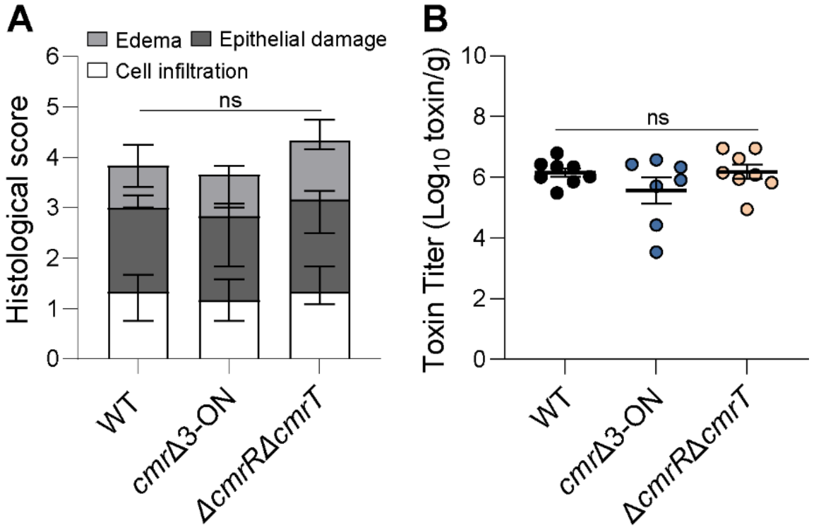

Figure S5. Histological score and toxin production in mice inoculated with WT or phenotypically locked mutants. (A) Histological scores for colons collected on day 2 p.i. Edema (light grey), epithelial damage (dark grey), and cell infiltration (white) were scored by a board-certified pathologist. No overall damage was observed for mock-inoculated animals. **(B)** Toxin levels in cecal contents. Toxin titers were calculated as the reciprocal of the highest dilution that caused ≥ 80% rounding of Vero cells. No cell rounding occurred when treated with cecal contents from mock-inoculated animals. Stacked bars **(A)** and lines **(B)** indicate medians, circles indicate individual animals, and error bars indicate the range. ns = not significant; Kruskal-Wallis test with Dunn's post-test.

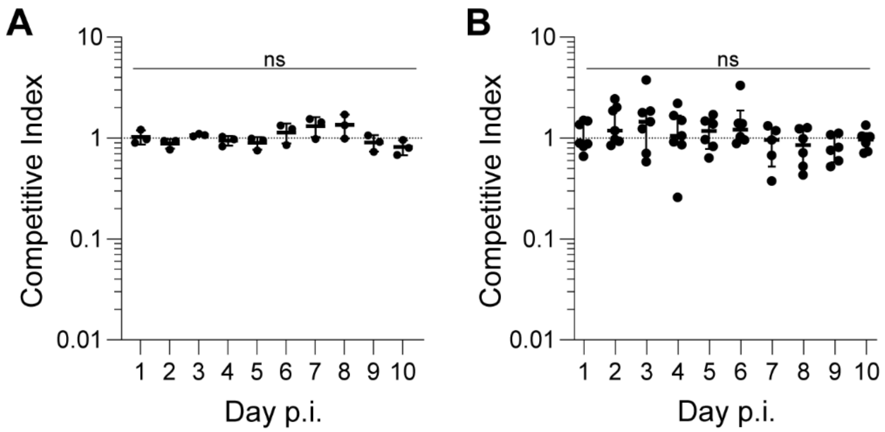

Figure S6. The *aad9* cassette does not affect the fitness of *cmr*Δ3-ON^R *in vitro* or of WT in mice. (A) *In vitro* competition. The *cmr*Δ3-ON^R and Δ*cmrR*Δ*cmrT* mutants were combined 1:1 and grown in TY broth; cultures were back-diluted and plated daily on BHIS-agar (total cells) and BHIS-agar with spectinomycin (*cmr*Δ3-ON^R cells) to determine CFU and the competitive index (CI). **(B)** *In vivo* competition. Mice were inoculated with 10^5 spores of spectinomycin-resistant (WT^R) and -sensitive (WT^S) wild-type *C. difficile*. Feces were collected daily and plated on TCCFA (total spores) and TCCFA with spectinomycin (WT^R spores only) to determine bacterial burden (CFU) and the CI. Circles represent values from biological replicate cultures (A) or individual mice (B), and lines indicate medians. ns = not significant; Wilcoxon rank sum test comparing values to a hypothetical CI of 1.

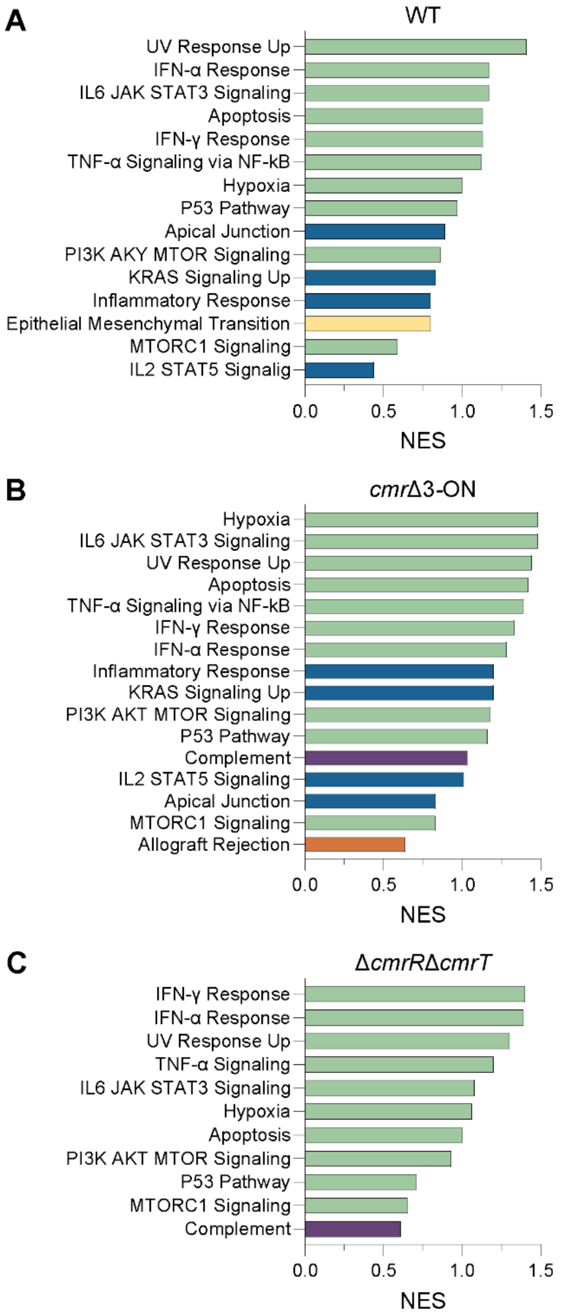

Figure S7. Gene set enrichment analysis. Normalized enrichment scores (NES) of pathways identified by GSEA of transcriptional data from mice inoculated with **(A)** WT, **(B)** *cmr*Δ3-ON or **(C)** Δ*cmrR*Δ*cmrT* at day 2 p.i. compared to uninfected mock controls. Green indicates enriched pathways shared among all infected groups; blue indicates pathways shared between WT or *cmr*Δ3-ON-infected mice; and purple indicates pathways shared between *cmr*Δ3-ON and Δ*cmrR*Δ*cmrT* infected mice. Yellow and orange indicate pathways enriched in mice infected with WT or cmrΔ3-ON, respectively.

Table S1. Strains used in this study.

Notation	Name	Description	Reference
RT273	R20291	*C. difficile* R20291 wild-type	1
RT2646	WT-R	rough colony variant isolated from R20291	2
RT2647	WT-S	smooth colony variant isolated from R20291	2
RT2406	*cmr*Δ3-ON	R20291 with the *cmr* switch locked in the ON orientation	2
RT2296	Δ*cmrR*Δ*cmrT*	R20291 with in-frame deletions on the *cmrR* and *cmrT* genes	2
	Escherichia coli DH5α	F- φ80*lacZ*ΔM15 Δ (*lacZYA*-*argF*) U169 *recA1 endA1 hsdR*17(rκ -, mκ+) *phoA supE44 thi*-1 *gyrA96 relA1* λ- *tonA*	3
RT270	*Escherichia coli* HB101(pRK24)	*E. coli* used in conjugations with *C. difficile*, Ap^R, Cm^R	4
RT2891	*E. coli* DH5α pRT2891	pMSR0 with CDR20291_2492-2493 homology arms and SphI site	This work
RT3098	*E. coli* DH5α pRT3098	pMSR0 with construct for *aad9* insertion at the CDR20291_2492 neutral site	This work
RT3371	*E. coli* DH5α pRT3371	pJB94 with construct for *aad9* insertion at the CDR20291_2492 neutral site	This work
RT3144	R20291 CDR2492::*aad9*	R20291 with spectinomycin-resistance cassette integrated between CDR20291_2492 and CDR20291_2493	This work
RT3480	R20291 *cmr*Δ3-ON CDR2492::*aad9*	R20291 *cmr*Δ3-ON with spectinomycin-resistance cassette integrated between CDR20291_2492 and CDR20291_2493	This work

Table S2. Primers used in this study.

Primer Number	Oligonucleotide (5' → 3')	Description
***C. difficile* primers**		
R2273	TCATTACCAGGTGTAGCAGTGAATGC	*rpoA* forward
R2274	GATAGAGCATGGTCCTTGAGCTTCT	*rpoA* reverse
R2270	GGAGATATATGGAGTTAGTGGTGCAA	*cmr*-ON forward
R2271	CTAGCCAATAGACAAGTTTCTAGAAAAATA	*cmr*-ON reverse/*cmr*-OFF forward
R2272	GAACAATTCTTGAATATTGTATTGAACATTAAGA	*cmr*-OFF reverse
R2914	CATTGATTTCTTTCAGTTTCGGATCCCTCCTGAAATACTTGATGAACCAGAAG	Amplification of CDR20291_2492 homology arms to generate pRT2891
R2917	GACGTCGACTCTAGAGGATCCTACATGCAATAACAGTACCTCAGGA	Amplification of CDR20291_2943 homology arm to generate pRT2891
R3192	GAGTGTCTCATGAACAAAAGTTCACGCATGCTTTGAGACTCTCTCTTTCATATCATATACC	Amplification of CDR20291_2943 homology arm to generate pRT2891
R3193	GGTATATGATATGAAAGAGAGAGTCTCAAAGCATGCGTGAACTTTTGTTCATGAGACACTC	Amplification of CDR20291_2492 homology arms to generate pRT2891
R3416	CAAAAGTTCACGCATGCTTCGAAGATAAAAAATTTAGAAGCC	Forward Gibson primer for *aad9* cassette amplification to clone in SphI site of pRT2891 generating pRT3098
R3417	GTCTCAAAGCATGCCCTAGGCATAAAAATAAGAAGCCTG	Reverse Gibson primer for *aad9* cassette amplification to clone in SphI site of pRT2891 generating pRT3098
R2987	GTATGTTAGAAGTTGTTTCAGAAGGC	Forward primer to screen for insertions between CDR20291_2942 and CDR20291_2943 in vector
R2988	GTGGCTGAAGTAGTATCAGAAGC	Reverse primer to screen for insertions between CDR20291_2942 and CDR20291_2943 in vector
R3472	GAGATAACATTAATTGGTAGTGGTACAAC	Forward primer to screen for chromosomal insertions between CDR20291_2942 and CDR20291_2943
R3473	CTGGACTGAACTTCAAGATAATTATG	Reverse primer to screen for chromosomal insertions between CDR20291_2942 and CDR20291_2943
R3951	CCGCGTCGACTAGTTCTTTAGCTCCTGAAATACTTGATGAACCAGAAG	Reverse primer to amplify the *aad9* cassette and flanking homology arms from pRT3098, adding an SpeI site, to generate pRT3371
R3952	CTGCAGGCCTCGAGTTATTTTACATGCAATAACAGTACCTCAGG	Forward primer to amplify the *aad9* cassette and flanking homology arms from pRT3098, adding an XhoI site, to generate pRT3371
**Murine primers**		
R3753	AGGAGCCAAGAGTGAAGAAC	*Tbp* forward
R3754	CTTCACATCACAGCTCCCCA	*Tbp* reverse

R3755	CCACCTTTTGACAGTGATGA	*Il1β* forward
R3756	CTGAAGCTCTTGTTGATGTG	*Il1β* reverse
R3757	GGGATTCACCTCAAGAACAT	*Cxcl1* forward
R3758	TTCTTTCTCCGTTACTTGGG	*Cxcl1* reverse
R3933	CTGGGAAATATGCACAGGTA	*Lcn2* forward
R3934	GTGAAACGTTCCTTCAGTTC	*Lcn2* reverse
R3937	ATCTTCTCAAAATTCGAGTGAC	*Tnfα* forward
R3938	AGTAGACAAGGTACAACCCA	*Tnfα* reverse

REFERENCES

1. Stabler R.A., He M., Dawson L., *et al*. Comparative genome and phenotypic analysis of *Clostridium difficile* 027 strains provides insight into the evolution of a hypervirulent bacterium. *Genome Biol*. 2009;10(9):R102. doi:10.1186/gb-2009-10-9-r102.
2. Garrett E.M., Mehra A., Sekulovic O., and Tamayo R. Multiple Regulatory Mechanisms Control the Production of CmrRST, an Atypical Signal Transduction System in *Clostridioides difficile*. *mBio*. 2021;13(1):e0296921. doi:10.1128/mbio.02969-21.
3. Hanahan D. Studies on transformation of *Escherichia coli* with plasmids. *J Mol Biol*. 1983;166(4):557-580. doi:10.1016/s0022-2836(83)80284-8.
4. McBride SM, Sonenshein AL. Identification of a genetic locus responsible for antimicrobial peptide resistance in *Clostridium difficile*. *Infect Immun*. 2011;79(1):167-176. doi:10.1128/IAI.00731-10.
5. Garrett EM, Sekulovic O, Wetzel D, et al. Phase variation of a signal transduction system controls *Clostridioides difficile* colony morphology, motility, and virulence. *PLoS Biol*. 2019;17(10):e3000379. doi:10.1371/journal.pbio.3000379
6. Sudarsan N, Lee ER, Weinberg Z, et al. Riboswitches in eubacteria sense the second messenger cyclic di-GMP. *Science*. 2008;321(5887):411-413. doi:10.1126/science.1159519
7. McKee RW, Harvest CK, Tamayo R. Cyclic Diguanylate Regulates Virulence Factor Genes via Multiple Riboswitches in *Clostridium difficile*. *mSphere*. 2018;3(5):e00423-18. doi:10.1128/mSphere.00423-18
8. Sekulovic, O., Mathias Garrett, E., Bourgeois, J., Tamayo, R., Shen, A., & Camilli, A. Genome-wide detection of conservative site-specific recombination in bacteria. *PLoS Genetics*. 2018;14(4), e1007332. doi.org/10.1371/journal.pgen.1007332
